# Micro-CT Analysis of Y-TZP Copings Made by Different CAD/CAM Systems: Marginal and Internal Fit

**DOI:** 10.1155/2018/5189767

**Published:** 2018-09-05

**Authors:** Rafaella Caramori Saab, Leonardo Fernandes da Cunha, Carla Castiglia Gonzaga, Amanda Mahammad Mushashe, Gisele Maria Correr

**Affiliations:** ^1^DDS, Graduate Student of the School of Health Sciences, Universidade Positivo, Curitiba, PR, Brazil; ^2^DDS, MSc, PhD, Professor of the School of Health Sciences, Universidade Positivo, Curitiba, PR, Brazil; ^3^DDS, MSc, Graduate Student of the School of Health Sciences, Universidade Positivo, Curitiba, PR, Brazil

## Abstract

**Purpose:**

The aim of the study was to measure the marginal and internal adaptation (MIA) of zirconia copings, made of 4 ceramic systems for CAD/CAM, using microcomputed tomography (micro-CT) technology.

**Material and Methods:**

Two identical stainless steel models were used, representing a preparation for full ceramic crown on a lower molar. The master models were then scanned for the production of copings from specific yttrium oxide partially stabilized tetragonal zirconia polycrystal ceramic blocks of each system (*n*=10): Cerec/inCoris Zi, Sirona; Cercon/Cercon base, Dentsply; Ceramill/Ceramill Zi, Amann Girrbach; and Lava/Lava Frame Zirconia, 3M ESPE. MIA was evaluated measuring 4 points as follows: marginal gap (MG), axial wall gap (AW), axio-occlusal angle gap (AO), and central occlusal area gap (CO). The data were statistically analyzed by ANOVA and Tukey's test (*α* = 0.05).

**Results:**

The ceramic system Lava showed greater internal desadaptation (80.75 ± 22.69 *µ*m) while CEREC presented the lowest values (49.92 ± 11.77 *µ*m). There were significant differences between the measurement points evaluated. CO demonstrated the greater values (77.03 ± 22.61 *µ*m). All marginal and internal adaptation values were considered clinically acceptable.

**Conclusion:**

It was concluded that there was an influence of the type of ceramic system in marginal and internal adaptation of copings in zirconia.

## 1. Introduction

The use of computer-aided technology (CAD/CAM) in the manufacture of ceramic restorations allows faster and more accurate indirect procedures [1, 2]. Among the various ceramic systems that can be machined by this method, the yttrium oxide partially stabilized tetragonal zirconia polycrystal (Y-TZP) is an aesthetic material with excellent mechanical properties, used mostly as infrastructures of unitary or partial fixed prosthesis.

The technique for milling zirconia in CAD/CAM uses a series of processing steps, such as milling and sintering, which can interfere with the accuracy of adjustment of the restoration [3]. Clinical longevity of indirect restorations is directly affected by the appropriate cervical sealing determined by the marginal and internal fit [4–6]. For an acceptable clinical protocol, restoration fit should provide a cement line thickness thinner than 100–120 *μ*m, ensuring minimal exposure of the cementing material to the buccal environment [7, 8] and, consequently, avoiding processes such as dissolution of the luting agent, decrease of bond strength, marginal staining, secondary caries, and pulp inflammation, among others. Likewise, internal gaps greater than 120 *µ*m may reduce the fracture strength of brittle materials, such as ceramics.

The computerized microtomography (micro-CT) has been applied for nondestructive analysis of marginal and internal fit of indirect restorations [9–12]. Through the difference in the electronic density of the materials exposed to the X-ray beams, the obtained images allow the evaluation in two or three dimensions of adaptation line in several cuts and directions, allowing a great number of measurement sites and facilitating the recognition of critical distances [4, 13, 14].

Thus, the objective of this study was to evaluate the internal and marginal fit of different presintered zirconia and CAD/CAM systems through micro-CT analysis. The null hypothesis to be tested is that there will be no difference between the systems tested and the measurement points evaluated.

## 2. Materials and Methods

Four commercial brands of CAD/CAM systems and presintered Y-TZP blocks were used for the preparation of specimens. The different CAD/CAM and ceramic systems and their respective manufacturers are described in Table 1.

In order to facilitate the micro-CT measurements, two identical stainless steel models simulating an inferior molar total ceramic crown abutment were constructed. The abutment features were as follows: 6° taper, 1.5 mm axial reduction, 2 mm occlusal reduction, and a supragingival chamfer finish line. After casting, the master models were polished with rubber tips for amalgam polishing (Viking, KG Sorensen, Barueri, SP, Brazil).

The master models were digitalized using the specific intraoral digitalizing device of each CAD/CAM system tested and the copings milled according to the respective manufacturer's instructions. The specifications of each software and milling unit used are described in Table 2. The blocks were machined according to the protocol defined in this study, which established marginal and internal cement spacer of 20 *µ*m and 70 *µ*m, respectively. The sintering process was carried out according to the manufacturer's instructions for each CAD/CAM ceramic material.

Micro-CT measurements of marginal and internal fit were performed by X-ray microtomography. The specimens were individually seated on the master model, and the set, composed of master model and coping, was scanned in a SkyScan 1172 micro-CT scanner (SkyScan, Aartselaar, Belgium). Images were acquired using 100 kV maximum accelerating voltage, 100 *µ*A current, and Al+ Cu filter with a pixel size of 13 *µ*mm. The specimens were scanned at frames per rotation step of 0.4 (180°).

After scanning, the images were reconstructed in software NRecon, which uses an FDK (Feldkamp–Davis–Kress) algorithm. The CT Analyser (CTAn) (SkyScan, Aartselaar, Belgium) software was used to obtain cuts from the coping central region in the mesial-distal and buccal-palatal directions. The files were saved as .bmp format.

After software processing of the dataset, the marginal and internal fit of the specimens were assessed in accordance with the following criteria: marginal gap (MG), axial wall (AW), axio-occlusal angle (AO), and central of occlusal area (CO). The measurement points were assessed in both sides of the model, and the mean value was recorded. ImageJ software was used to perform the measurements by a single-calibrated examiner (ImageJ, US National Institute of Health, Bethesda, Maryland, USA).

Results from different measuring points were analyzed statistically using two-way (ceramic system and measurement points) analysis of variance and Tukey's test, at a significance level of 5%.

## 3. Results

The means and standard deviations for the evaluated points according to the CAD/CAM systems are shown in Table 3.

The results indicated statistically significant differences for the material factors (*p* < 0.00001) and points (*p* < 0.00001) and for the material interaction points (*p*=0.000027).

For the material factor, the LA ceramic system showed significantly higher marginal and internal gap, on the following sequence: LA > CN = CL > CC.

Considering the evaluation points, CO presented the highest values of gap, as follows: CO > AO > AW > MG.

## 4. Discussion

In order to reduce the excessive exposure of the cement line, and consequently its degradation, and to avoid the mechanical weakening of the ceramic restoration, it is recommended that the marginal and internal gap of the restorations be less than 120 *μ*m [7, 8]. In the present study, all the systems and measurement points tested presented values less than 110 *μ*m and were considered, therefore, clinically acceptable. A possible hypothesis for such performance refers to the use of an anatomical format for the master model abutment. Habib et al. [14] verified that the marginal and internal adaptation of zirconia copings with this type of preparation presents better fit when compared to nonanatomical preparations.

Despite the good performance of the tested groups, there was a significant difference between the evaluated points and between the CAD/CAM systems, thus rejecting the null hypothesis.

In agreement with similar studies [15, 16], the analysis by micro-CT showed an increase in the thickness of the cementation line in the margin-center of the occlusal area direction. The axio-occlusal angle and the occlusal surface, due to their inherent anatomical detail, may be more subject to imperfections related to the preparation, thus determining a worse fit. The higher surface irregularity of this region may, in the same way, negatively influence the light reflection during the preparation scan [16]. Thus, image capture and construction may have lower fidelity, increasing the level of maladaptation of the restoration.

In this study, there was a statistical difference between the ceramic systems for CAD/CAM, being that the CC group had the best adaptation values, independently of the evaluated point. Unlike the other systems tested, the technology applied to the CC system uses irrigation during the manufacturing of the restoration, which may influence the higher performance related to coping fit. Irrigation is recommended for blocks with a high degree of sintering, in order to avoid heating and block damage during the machining process [17]. Thus, the cooling of the system allows the achievement of a high degree of sintering, with low risk of distortion, providing greater fidelity to the final restoration [18].

The results of this study showed that the LA system presented the worst levels of adaptation, a finding also presented in similar studies [19, 20]. Regarding the performance between CC and CN, Shim et al. [20] did not find any difference of fit between the systems, diverging from the present study which showed better fit values for CC.

In the present study, it was decided to use presintered ceramic blocks, since they have a faster machining process and less wear of the milling units, increasing their useful life. The shape, diameter, and size of cutters can directly influence the accuracy of the restoration, with smaller units tending to be machined with better detail and fidelity, for example, to define the AO angle with more precision.

In addition to the type of tip used, the fit fidelity of each system can be determined by the number of axes during machining. In this work, each CAD/CAM system had a different number of axes (CC: 2, LA: 3, CN: 4, and CL: 5) (Table 2). Beuer et al. [17] stated that the quality of the adaptation does not necessarily increase the number of axes. This fact is in agreement with the results found in this study, where it was demonstrated that the CC system, which presents the smallest number of axes, obtained better performance.

Several aspects were standardized on this study that enables the comparison between the different points and systems and to reduce possible confusion variables. In this study, the application of coating on copings was eliminated, eliminating the effects related to burning, application time, and interaction between ceramic and glaze types [21, 22].

Other parameters and clinical studies are still needed to confirm whether the adaptation between different systems is really similar and clinically verify the longevity of these ceramic CAD/CAM systems.

## 5. Conclusion

It can be concluded that there was influence of the type of ceramic system in the marginal and internal adaptation of copings in zirconia. The marginal adaptation presented the smallest dimension evaluated while the center/occlusal adaptation presented the greatest gap. The adaptation values were within the clinically acceptable.

## Figures and Tables

**Table 1:** Group distribution and material specification (*n*=10).

Group	CAD/CAM system	Ceramic system	Manufacturer	Ceramic system composition
CC	CEREC	In Coris Zi	Sirona Dental Systems, Bensheim, Germany	Zirconium dioxide + hafnium dioxide + yttrium trioxide >99 wt.%, aluminum trioxide <0.5 wt.%, others oxides <0.5 wt.%
CN	CERCON	Cercon base	Dentsply International, Susquehanna Commerce Center, York, USA	Yttrium trioxide 4.5–5.4 wt.%, hafnium dioxide <5 wt.%, aluminum trioxide <0.5 wt.%, silicon dioxide <0.1 wt.%, sodium oxide <0.1 wt.%, iron trioxide <0.1 wt.%, zirconium dioxide balance
CL	CERAMILL	Ceramill Zi	Amann Girrbach North America, Charlotte, USA	Zirconium dioxide + hafnium dioxide + yttrium trioxide >99 wt.%, yttrium trioxide 8.5–9.5 wt.%, hafnium dioxide <5 wt.%, aluminum trioxide <0.5 wt.%, other oxides <1 wt.%
LA	LAVA	Lava Frame Zirconia	3M, St. Paul, USA	Zirconium oxide 79–97%, yttrium oxide 3–15%, hafnium oxide <5%

**Table 2:** Software and milling specifications for each CAD/CAM system.

Group	Software	Milling machine	Number of axes	Milling tools/burs
CC	CEREC SW 4.2.4	inLab MC XL CEREC	2	Cylinder pointed bur 12S and 12 EF and cylinder bur 12 EF
CN	CERCON ART 3.2	CERCON Brain	4	Parallel burs 10.0 and conical burs 4°
CL	Ceramill mind	Ceramill Motion 2	5	Ceramill Roto Motion 0.6; 1.0 and 2.5
LA	LAVA CAD	LAVA Form Milling Unit	3	Lava milling bur types 4 and 5

**Table 3:** Mean (*µ*m) and standard deviation (SD) values for the different groups after fit measurement (*n*=10).

Points	CC	CN	CL	LA
MG	37.68 ± 9.04^a^	57.40 ± 8.71^bcd^	52.50 ± 10.18^abc^	62.70 ± 11.11^bcd^
AW	47.50 ± 9.00^ab^	61.93 ± 15.37^bcd^	55.48 ± 7.79 ^bc^	69.05 ± 10.54^cde^
AO	54.53 ± 8.49^abc^	56.52 ± 10.16^bcd^	62.68 ± 9.72^bcd^	82.40 ± 16.65^e^
CO	59.98 ± 7.53^bcd^	73.40 ± 12.98^de^	65.90 ± 8.04^cde^	108.85 ± 18.01^f^

Values with different superscript letters are significantly different (Tukey *p* > 0.05). CC, Cerec; CN, Cercon; CL, Ceramill; LA, Lava; MG, marginal gap; AW, axial wall; AO, axio-occlusal angle; CO, central of occlusal area.

## Data Availability

The data used to support the findings of this study are included within the article.
